# Teacher Training Can Make a Difference: Tools to Overcome the Impact of COVID-19 on Primary Schools. An Experimental Study

**DOI:** 10.3390/ijerph17228633

**Published:** 2020-11-20

**Authors:** Teresa Pozo-Rico, Raquel Gilar-Corbí, Andrea Izquierdo, Juan-Luis Castejón

**Affiliations:** Department of Developmental Psychology and Didactics, University of Alicante, 03080 Alicante, Spain; aig18@alu.ua.es (A.I.); jl.castejon@ua.es (J.-L.C.)

**Keywords:** teacher stress, burnout, information and communication technologies, emotional intelligence, teacher continuing education, primary school

## Abstract

Overcoming the impact of the coronavirus pandemic (COVID-19) on primary schools is an emerging need and priority in the current social welfare system. Accordingly, this study presents an empirical learning package to support teachers, who perform frontline work in schools, in coping with stress, preventing burnout, improving their information and communications technology (ICT) competency, and introducing the principles of emotional intelligence (EI) in the classroom. The participants included 141 primary school teachers (M = 38.4 years, SD = 6.84; 54.6% women). They were randomly assigned to an experimental or control group. The experimental group participated in the 14-week teacher training program, whereas the control group did not participate in the program or receive any other training during the intervention. Repeated-measures analysis of variance (time x group) was performed to identify the effects of the teacher training program. Teachers who participated in the training program evaluated it positively and showed significant differences compared to the control group in their abilities to cope with stress and avoid burnout, their ICT competency, and their introduction of EI in the classroom. Implications for supporting teachers are discussed.

## 1. Introduction

This research aimed to demonstrate the efficacy of a teacher training program intended to support primary school teachers in four key areas: (1) coping with stress, (2) preventing burnout, (3) improving their information and communications technology (ICT) competency, and (4) introducing emotional intelligence (EI) in the classroom. Coronavirus disease 2019 (COVID-19) is having a clear impact on the educational setting of primary school; practical teaching strategies and methodologies are required to face and overcome this new threat to health, social development, and education. The following subsections describe the current state of research in this field and the teacher training program

### 1.1. Theoretical Framework

#### 1.1.1. Work-Related Stress among Teachers

Several studies have demonstrated a positive relationship between teachers’ well-being and their efficacy in teaching [1,2,3,4,5,6,7,8,9,10,11,12,13,14,15]. However, practical and applied research on training programs to improve teacher well-being and efficacy is still needed, particularly in light of the extreme challenges posed by the COVID-19 crisis. Furthermore, even if teachers do receive training in educational methodologies to manage stress, classroom-level implementation can remain low [16,17].

It is important to consider in order to support and design efficient training programs like this and according to previous researches, that the resources that can mitigate or reduce teacher stress and burnout are internal (managing classroom self-efficacy and instructional self-efficacy) and external (school support resources). Both (internal and external resources) have shown a negative effect on job stressors [18]. For example, as Doménech-Betoret and Gomez-Artiga [19] pointed out, there is a strong and significant association between self-efficacy and the coping strategies used by teachers, and coping strategies act as moderator on burnout dimensions.

Appropriate training can mitigate work-related stress among teachers by furnishing them with the skills, strategies, and resources needed to manage stress, improve efficacy, and increase workplace satisfaction [20,21,22,23]. The key to the effectiveness of such training is to provide teachers with useful strategies and resources and facilitate effective transfer of these skills to the classroom to buffer against teacher stress in everyday school life, as the program developed in the present study does.

On this way, teacher burnout is a serious problem in schools, with links to impoverishment of the teaching occupation, frustration and dissatisfaction with teaching, and job absenteeism [24,25,26,27,28,29,30]. It is critical to train teachers to face this difficulty, safeguard themselves, and achieve optimal psychological development at work [31,32,33,34,35].

Training to prevent teacher burnout plays a protective role and improves the psychosocial environment and health of teachers’ work life [36,37,38,39,40]. It also increases teachers’ engagement, resilience, perception of their teaching value, self-efficacy, and ability to thrive within their [41,42,43,44,45,46]. Thus, a positive relationship exists between teachers’ well-being and their efficacy in teaching [47,48,49].

Similarly, preventing teacher burnout has been linked to decreases in disruptive behavior among students and greater general stability of the classroom [50,51,52], as well as student motivation and academic commitment [53,54,55,56]. Teachers with low stress levels and no burnout symptoms and classes with high coping skills have been associated with enriched student outcomes [57,58,59,60,61,62].

#### 1.1.2. ICT Educational Approaches

Providing a teacher training program in the ICT area to reduce the impact of COVID-19 is considered important because ICT has become a key resource in 21st-century education, particularly during the COVID-19 outbreak, during which face-to-face classes have not been possible [63,64,65,66,67,68]. Several studies have shown that introduction of ICT in teaching and learning improves the quality of education [69,70,71,72]. However, incorporating ICT into education also places significant professional demand on teachers [73,74,75,76]. Thus, adequate teacher training on practical ICT competency geared toward the teaching/learning process is necessary [77,78,79,80]. Without such training, forcing the introduction of ICT educational approaches into the curriculum and assessment process may cause considerable frustrations to both teachers and the learning community [81,82].

#### 1.1.3. EI for Educational Development

Several studies have shown that teachers require EI competency to support student learning, provide opportunities for social development, and promote academic achievement and success among students [83,84,85,86]. Similarly, EI competency can increase teachers’ own work environment, psychosocial health, and well-being [87,88,89,90,91,92,93,94,95,96,97]. Both applications have health benefits and positive impacts on the learning community.

Developing teachers’ abilities to cope with negative affect and emotional exhaustion is key in an academic context, as it has a meaningful effect on learning processes in the classroom setting and plays a significant role in fostering students’ learning engagement [77,98,99,100,101,102]. It also plays a protective role among teachers, increasing the quality and health of teachers’ psychosocial environment and working life [103,104,105,106].

Finally, emotional exhaustion plays a key role in teacher burnout [18]. For this reason, providing a teacher training in the EI area is considered important in order to prevent emotional exhaustion and, consequently, to reduce the negative impact of COVID-19.

## 2. Materials and Methods

### 2.1. Participants

The request for participation in the study was sent to 500 teachers from three educational districts in the southeast of Spain. A total of 323 primary school teachers showed interest in participating voluntarily in the study. However, for the present study only those teachers who demonstrated a prior ICT-related training experience (an ICT course of 30 hours achieved in the academic year before) were selected (*n* = 141). Consequently, these 141 primary school teachers were randomly assigned to one of two experimental conditions. The first (experimental) group (*n* = 70) participated in the teacher training program intended to improve stress management and burnout in the teaching profession, competency and use of ICT to support teaching and learning, and introduction of EI-based pedagogical principles into the classroom. The second (control) group (*n* = 71) did not receive any special training in strategies for managing stress or burnout, using ICT to support teaching and learning, or introducing EI into their teaching. Of the 141 teachers, 54.6% were women and 45.4% were men, and their average age was 38.4 years (SD = 6.98 years). Average teaching experience was 13.1 years (SD = 6.84 years). Finally, the procedure was approval for University of Alicante Ethics Committee (UA-2015-07-06).

### 2.2. Instruments

The following instruments were used in this study to measure teacher stress, burnout, emotional intelligence, and evaluation of the teacher training program.

1. Perceived Stress Questionnaire (PSQ, 20-item) [107]: The PSQ evaluates subjective experience of perceived stressful situations [105]. The scale includes 20 items (e.g., “You have many worries”) that are formulated positively and negatively to reduce acquiescence bias, and each item is assessed on a 4-point Likert-type scale from 1 (“almost never”) to 4 (“almost always”). It includes six subscales in addition to the overall score: (1) Harassment–social acceptance, (2) Overload, (3) Irritability–tension–fatigue, (4) Energy–joy, (5) Fear–anxiety, and (6) Self-realization–satisfaction. Higher scores indicate more severe subjective experience of perceived stress. Cronbach´s α values were optimal (between 0.85 and 0.87). The PSQ has been translated and validated in several languages and cultural contexts; the Spanish version was used in the present study [108].

2. Perceived Stress Scale (PSS, 14-item) [109] The PSS is a self-reported instrument that assesses level of perceived stress during the last month and consists of 14 items (e.g., “In the last month, how often have you felt that you were unable to control the important things in your life?”) rated on a 5-point scale from 0 (“never”) to 4 (“very often”). Higher scores indicate higher perceived stress. The instrument was originally designed in English [109] and has been translated and validated in several languages and cultural contexts. The European Spanish version was used in the present study and showed adequate reliability (internal consistency, α = 0.81; test-retest, r = 0.73), validity (concurrent), and sensitivity [110].

3. RED questionnaire [111]: The RED questionnaire assesses psychosocial labor risks and workplace stress due to increasingly heavy technological demands or insufficient technological competency. The scale includes 16 items rated on a 7-point scale from 0 (“never”) to 6 (“always/every day”) (e.g., “When I finish working with ICT, I feel exhausted”), and includes four subscales in addition to the overall score: (1) Skepticism, (2) Fatigue, (3) Anxiety, and (4) Inefficiency. Higher scores indicate that participants report feelings of anxiety, fatigue, skepticism and inefficacy beliefs related with the use of ICT at the workplace. The RED questionnaire was originally developed and validated for the Spanish population and demonstrates adequate reliability and validity (Cronbach’s α values were 0.93 for the Skepticism dimension, 0.92 for the Fatigue dimension, 0.83 for the Anxiety dimension, and 0.84 for the Inefficiency dimension).

4. Maslach Burnout Inventory (MBI; 22-item) [112]: The MBI includes 22 items presented as statements about the feelings and attitudes of the professional in his or her work (e.g., “I feel like I’m at the end of my rope”). It is used to measure both the frequency and intensity of professional burnout. Each item is rated on a 7-point Likert-type scale from 0 (“never”) to 6 “every day”). The instrument includes three subscales: (1) Emotional exhaustion or exhaustion, (2) Depersonalization, and (3) Personal accomplishment. Burnout syndrome is defined by high scores in the first two subscales and low scores in the third. The MBI demonstrates adequate reliability and validity (Cronbach’s α values were 0.89 for the Exhaustion or emotional exhaustion dimension, 0.77 for the Depersonalization dimension, and 0.74 for the Personal accomplishment dimension). This study used a version of the scale that has been adapted and validated for the Spanish population [113].

5. Emotional Quotient Inventory (EQ-i; 51-item) [23]: The EQ-i is a self-reported measure of emotionally and socially intelligent behavior. It has been translated into more than 30 languages and demonstrates good reliability and validity. The scale consists of 51 items (e.g., “I believe in my ability to handle most upsetting problems”) assessed on a 5-point Likert-type scale, and evaluates five subfactors of emotional intelligence in addition to the overall EQ-i score: (1) Intrapersonal intelligence, (2) Interpersonal intelligence, (3) Adaptation, (4) Stress management, and (5) Humor. This study used the Spanish version of the EQ-i [114]. Cronbach’s α values and internal consistency reliability were 0.75 for the Intrapersonal intelligence dimension, 0.77 for the Interpersonal intelligence dimension, 0.84 for the Adaptability dimension, 0.83 for the Stress management, and 0.88 for the Humor dimension.

6. Teacher training evaluation survey: A survey was designed ad-hoc by the authors to determine how satisfied teachers were with the 14-week training program after their participation. The survey consisted of 15 statements rated on a 5-point Likert scale from 1 (“totally disagree”) to 5 (“totally agree”). A comment box was also included to allow teachers to share remarks or suggestions. Please see Appendix A for more information.

### 2.3. Procedure

Before the training was initiated, all the teachers were fully informed of the study’s details and guaranteed confidentiality of all data obtained. On this way, the study was conducted in accordance with the Declaration of Helsinki and the recommendations and approval of the University of Alicante Ethics Committee (UA-2015-07-06). Participating teachers were randomly assigned to either the experimental or the control group. The experimental group participated in the training program over the next 14 weeks. The control group did not participate in the program or receive any other intervention during this period. In addition, on the one hand, the pretest measures were recollected two weeks before of the beginning of the program in both groups. For this goal, an e-learning Moodle platform was used because all the Spanish population was confined. On the other hand, the posttest measures were recollected two weeks after of the completion of the program in both groups. Also, in this case, the same e-learning Moodle Version 3.9.2 (Moodle.org, Western, Australia) platform was used. In conclusion, in both groups, teachers’ scores in all variables (stress levels, burnout levels, ICT competency, and EI competency) were recorded before and after the 14-week teacher training program. The experimental group also completed the teacher training evaluation survey after the training across the e-learning Moodle platform mentioned after conclusion of the training period.

### 2.4. Experimental Design and Data Analysis

An experimental study was carried out in this project with two groups (control vs experimental group) and two times of evaluation (pretest phase carried out before the training and the posttest phase carried out after the training). On this way, a general linear model was used to analyze the effects of the 14-week teacher training program. To ensure the equivalence of the groups, comparative analysis using Student’s *t*-test was conducted before initiating the teacher training program. Subsequently, teachers’ scores on all variables were analyzed using multivariate analysis of variance (MANOVA) and a univariate analysis of variance (ANOVA) of repeated measures, in which the dependent variable measured before and after the training was treated as intra-subjects variable, and the group (control/experimental) was an inter-subject variable.

Finally, the differences between the experimental and control groups were graphically represented for the variables studied. All statistical analyses and graphical representation were completed using SPSS version 22 (IBM Corporation, Armonk, NY, USA).

### 2.5. Teacher Training Program

First of all, the specific research objectives of the study are the following:

1. To analyze if there is a significant decrease in the stress levels of teachers in the experimental group compared to those in the control group after training.
2. To analyze if there is a significant decrease in the emotional exhaustion levels and depersonalization levels of teachers in the experimental group compared to those in the control group after training.
3. To analyze if there is a significant increase in personal accomplishment levels of teachers in the experimental group compared to those in the control group after training.
4. To analyze if there is a significant increase in emotional intelligence levels of teachers in the experimental group compared to those in the control group after training.
5. To analyze if there is a significant increase in use of ICT among teachers in the experimental group compared to those in the control group after training.
6. To analyze if there is a teacher’ satisfaction with the program considered like a effective training for prevented burnout and promoted psychological well-being in the teaching community.

Consequently, the present study’s 14-week teacher training program was designed to improve the key strategies described in Section 1.1.1 through Section 1.1.3 among primary school teachers. Table 1 describes the details of each lesson in the program.

## 3. Results

### 3.1. Baseline: Before Teacher Training

The study first analyzed whether the experimental and control groups had any significant differences in (1) stress levels (PSQ and PSS), (2) burnout levels (MBI), (3) ICT competency (RED questionnaire), (4) EI competency (EQ-i), or (5) sociodemographic variables (age, teaching experience, and gender) based on an independent-samples *t*-test. The results showed no significant differences between the two groups in any of the variables before the teacher training program (Table 2).

### 3.2. Effects of the Teacher Training Program

Box’s M test revealed no homogeneity of the variance-covariance matrix for all scales. However, violation of this assumption has a minimal effect if the groups are approximately equal in size [115]. 

Analyzing the effects of the teacher training program, multivariate analysis indicated that all the variables showed significant variations in interaction test, obtaining the following results for the Wilks’s lambda statistic: Harassment–social acceptance (F = 147.98, η^2^partial = 0.52), Overload (F = 114.57, η^2^partial = 0.49), Irritability–tension–fatigue (F = 166.32; η^2^partial = 0.55), Energy–joy (F = 119.17, η^2^partial = 0.46), Fear–anxiety (F = 115.55, η^2^partial = 0.45), Self-realization–satisfaction (parallelism F = 112.91, η^2^partial = 0.45), and PSQ total (parallelism F = 188.18, η^2^partial = 0.57); PSS total (F = 270.49, η^2^partial = 0.66); Skepticism (parallelism F = 191.88, η^2^partial = 0.58), Fatigue (F = 255.93, η^2^partial = 0.65), Anxiety (F = 353.06, η^2^partial = 0.72), Inefficiency (parallelism F = 350.36, η^2^partial = 0.72), and RED questionnaire total (F = 359.69, η^2^partial = 0.72); Emotional exhaustion (F = 238.85, η^2^partial = 0.63), Depersonalization (F = 444.53, η^2^partial = 0.76), and personal accomplishment (F = 117.82, η^2^partial = 0.46); Intrapersonal intelligence (F = 133.79, η^2^partial = 0.49), Interpersonal intelligence (F = 134.05, η^2^partial = 0.49), Stress management (F = 264.36, η^2^partial = 0.65), Adaptation (F = 258.80, η^2^partial = 0.65), Humor (F = 258.99, η^2^partial = 0.65), and EQ-i total (F = 223.87, η^2^partial = 0.62). The F ratios in all cases were highly significant, *p* ≤ 0.001, and the observed power was 1.00 and all cases.

Univariate analysis showed that, as shown in Table 3, the interaction between the evaluation time (pre-test and post-test) and the training is significant (*p* ≤ 0.001) for all the variables examined in the present study.

**Figure 1:**
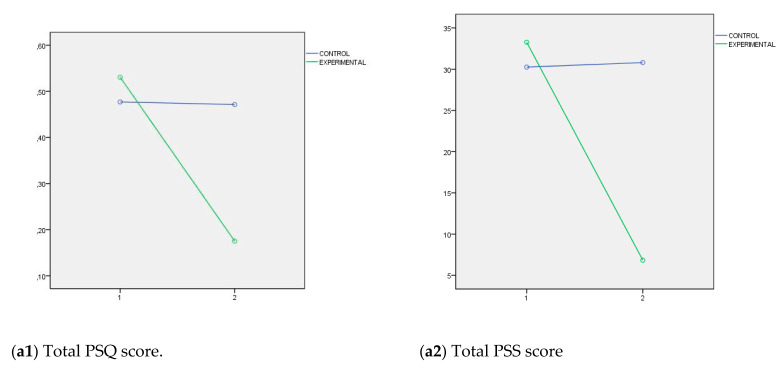

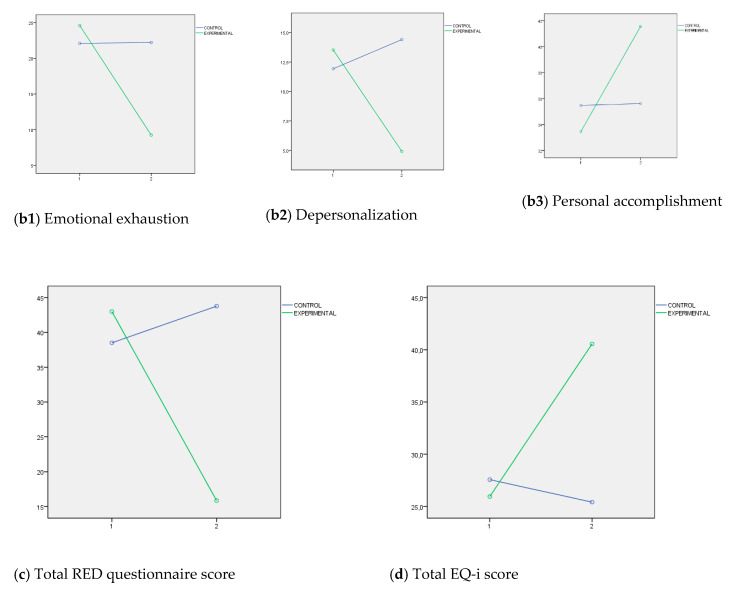
Estimated marginal means for (**a1**) total PSQ scores and (**a2**) total PSS scores (stress levels); (**b1**) Emotional exhaustion scores, (**b2**) Depersonalization scores, and (**b3**) Personal accomplishment scores on the MBI (burnout levels); (**c**) total RED questionnaire scores (ICT competency); and (**d**) total EQ-i scores (EI competency).

In order to simplify the graphic representations, Figure 1 shows interaction graphs of the direction of the differences observed between the experimental and control groups in the total score of PSQ, PSS, RED questionnaire and EQ-i. Furthermore, in the case of MBI, three factors are considered: Emotional exhaustion, Depersonalization, Personal accomplishment, because this instrument do not have a total scale score. After the 14-week training period, significant changes were observed in experimental group compared to the control group in terms of stress level (decreased total PSQ and PSS scores also represent decreases in all PSQ subscales), burnout levels (decreases in Emotional exhaustion and Depersonalization scores and increase in Personal accomplishment scores on the MBI), ICT competency (decreased total RED questionnaire scores also represent decreases in all RED subscales), and EI competency (increased total EQ-i scores also represent increases in all EQ-i subscales).

Finally, the experimental group showed high agreement with nearly all statements on the satisfaction survey, indicating high satisfaction with the teacher training program. An exception was item 6 (“The objectives were appropriate for the planned duration and the established work schedule”), which received a positive (“agree” or “strongly agree”) response from 78.9% of the participants but a negative (“disagree”) response from 21.1%. Responses to the open-ended comment box indicated that this was because some teachers would have liked the training to last longer.

## 4. Discussion

### 4.1. Interpreting the Findings

First of all, according with the first specific research objective, participants in the training showed a decreased in the stress levels. Second, according with the second and third specific research objective, participants in the training showed a decreased emotional exhaustion and depersonalization and an increased sense of personal accomplishment. Third, according with the fourth specific research objective, the participants in the training showed an increase in the emotional intelligence levels. 

Fourthly, according with the fifth specific research objective, the teacher training program included training on the use of ICT to overcome adverse educational and social-sanitary conditions; the association of ICT methodologies with key concepts and educational innovations applied to classroom challenges; ICT-based pedagogical methodologies for lesson planning and evaluation; and ICT-based innovations and game-based e-learning methodologies to foster a high-quality teaching/learning environment in primary education. On this way, the experimental group in this study demonstrated increased levels of perceived competency and perceived ability to deal with psychosocial labor risks and workplace stress as a result of the heavy and increasing technological demands imposed by COVID-19. This indicates that after participating in the training, teachers reported less anxiety, fatigue, skepticism, and inefficacy related to using ICT in their work. Considering these results, ICT in education can facilitate educational innovations to face classroom challenges and offers customizable tools to meet teachers’ instructional needs, particularly in the face of adverse educational and social-sanitary conditions.

On the other hand, according with the sixth specific research objective, in the post-training survey, participants strongly agreed that the training program effectively improved teachers’ ICT competency. Participants also agreed with other statements related to ICT and the set of competencies acquired in the training program, such as “the knowledge that I have gained will positively affect my methods in the classroom” and “this teacher training program was a good impetus for me to improve my performance as a teacher.” Finally, the study’s important goal of ensuring transfer of the knowledge gained in the training program in actual teaching practice was evidenced by participants’ strong agreement with the statement “I consider the transfer of knowledge to my teaching practice understandable.”

### 4.2. Theoretical and Practical Implications of the Study

The four variables examined in this study and addressed by the teacher training program have long been recognized as important issues in teaching and are even more key given the current difficulties posed by COVID-19 in primary education. Teaching is an occupation with high stress and emotional demands [88,116,117,118]. This study’s teacher training was found to effectively decrease teachers’ perceived stress levels and increase their EI. The evaluation survey revealed that teachers who participated in the program found it helpful in developing these two competencies and implementing this knowledge in the classroom (e.g., participants reported strong agreement with statements such as “the training program effectively improves teachers’ ability to cope with stress” and “the training program effectively trains teachers to apply EI competencies to educational management”).

This study aimed to incorporate a practical approach. Thus, the teacher training program incorporated practical tools, methodologies, and resources to help teachers manage their stress in the classroom, both in normal educational contexts and in adverse social and health conditions; improve their understanding of emotions (academic self-esteem, self-realization, and emotional awareness), ability to identify emotions (improving intrapersonal and interpersonal relationships, increasing empathy and social responsibility in crisis situations), emotional expression, and ability to leverage positive emotions; implement EI strategies and improve their effectiveness in frontline teaching work; and promote positive emotions in educational plans to face and resolve challenges. In the post-training survey, participants strongly agreed that the teacher training program was helpful for developing innovative and successful teaching methodologies and educational plans as a strategic response to address the pandemic. Furthermore, training and improving these competencies are key for teaching because managing stress and applying EI in the classroom are linked to favorable school policies and practices [119,120,121,122,123,124,125,126,127,128,129,130].

Preventing teacher burnout is also essential for optimal teacher effectiveness [131,132,133,134,135,136]. Burnout syndrome appears due to chronically high levels of stress, overwhelming negative emotions, and sustained work-related exhaustion. The teacher training program in this study incorporated methodologies aimed to prevent burnout along with practical strategies and resources to safeguard psychological well-being in situations of adversity and health emergency. 

Training in ICT competency was included in this study’s teacher training program for several reasons. First, the scientific literature shows that ICT competency is an emerging requirement for innovative and high-quality teaching [137,138,139,140,141,142,143,144,145]. Second, methodologies based on ICT are useful educational tools valid path towards the teaching [146,147,148,149,150]. Third, creative educational approaches are needed to mitigate the impact of COVID-19 on the learning community [151,152,153]. Finally, workable solutions are required to cope with health situations that prevent or restrict face-to-face teaching [154,155,156]. 

### 4.3. Limitations and Future Research Directions

This study presents a practical training program to improve teacher competencies and help them mitigate the impact of the COVID-19 crisis on primary education. However, several limitations must be considered. First, the sample size was small. A future goal is to develop an improved version of this training program and implement it with a larger sample of teachers. Additionally, the initiative is small in scope. Unfortunately, there is currently no global or standard educational response to the COVID-19 pandemic; each country is addressing the crisis based on their own opportunities and educational expertise. In future, the scope of the training should be improved to adapt the goals and methodologies to international educational contexts.

Second, in the light of the study’s results, future research must confirm the long-term sustainability of the methodologies trained in the program and their impact on students’ academic achievement. Although this study intended to analyze academic achievement reports, educational authorities have strongly promoted positive academic reports during the pandemic, and thus academic reports published during the COVID-19 outbreak cannot be considered accurate.

Finally, transfer of the methodologies acquired in the teacher training program requires time, and logistical and pedagogical considerations must be guaranteed. The study found that the program’s strategies effectively improved teachers’ ability to cope with stress and avoid burnout, but teacher overload should be realistically considered, taking into account the time and resources that are available. Similarly, knowledge from the teacher training program could be transferred to implement meaningful EI learning into the classroom and promote a free and constructive environment, but a school culture of respect between the members of the learning community is necessary for this to succeed. Finally, the ICT training for teachers in the program is accurate, but the digital gap must still be fought. The entire educational community must have the technical resources and skills to access classes designed in an e-learning environment. For the proposed methodologies to be viable, these educational conditions must be met before teacher training is implemented. Further research on these aspects is necessary.

## 5. Conclusions

The challenges of the COVID-19 health situation in education have led to the emergence of a teacher overexertion and a depth adaptation to the new environment demands on classroom. Numerous questions have been raised about the short- and long-term impacts of the COVID-19 pandemic on the Primary Education. New demands on current education systems are extensive and have developed suddenly and unpredictably. However, there is consensus that training teacher competencies is key in responding to the pandemic situation. The purpose of this study is to develop a teacher training for support teachers, who perform frontline work in schools, in coping with stress, preventing burnout, improving their information and communications technology and introducing the principles of emotional intelligence in the classroom. Accordingly, the results have shown that fostering teachers’ well-being (through stress management and prevention of burnout) is possible, as well as equipping teachers with the ICT competency necessary to implement new educational practices and incorporating EI into teaching. And consequently, raising teachers’ ability to strategically create a positive emotional atmosphere within the learning community.

## Figures and Tables

**Table 1:** Teacher Training Program.

WEEK	TOPIC	METHODOLOGY
1	Presentation of the teacher training program and description of its four key strategies: (1) coping with stress; (2) preventing burnout; (3) improving ICT competency; (4) improving EI competency.	E-learning discussion forum on the topics of the training and their implementation in the classroom.
2	Teaching methodologies to manage stress and prevent burnout in educational contexts during adverse social/health circumstances: practical teacher tools.	Practical activity involving real implementation of practical teacher tools (learned in the training) into classroom teaching, and discussion of results obtained as a strategic response to the pandemic.
3	The possibilities offered by ICT in educational contexts for overcoming adverse educational and socio-sanitary conditions.	E-learning circle discussion of the usefulness of ICT for teachers in fighting the psychological and academic impacts of COVID-19 on the educational community.
4	Principles of ICT methodologies and their association with key concepts and educational innovations applied to classroom challenges.	Case studies on the pedagogical design of ICT integration and technological pedagogical content knowledge to reducing workloads and transform the educational process.
5	Lesson planning and evaluation strategies based on ICT methodologies: pedagogical practices given a set of adverse conditions.	Incorporating problem-based, project-based, and inquiry-based learning and design thinking into teaching plans as common pedagogical practices applied to classroom challenges.
6	ICT-based innovations and game-based e-learning methodologies to promote a high-quality teaching/learning environment in primary education	E-learning infographics about successful implementations of approaches curriculum changes based on game-based learning methods and strategies.
7	Understanding emotions: practical teacher tools to improve academic self-esteem, self-realization, and emotional awareness in adverse conditions.	Educational teacher practice based on implementing EI into teaching to improve understanding of students’ motivation, learning, and performance.
8	Identifying emotions: practical teacher tools to improve intrapersonal and interpersonal relationships, empathy, and social responsibility in crisis scenarios.	Designing learning environments to foster high EI in the classroom (e.g., guidance in communication and relationships to create a positive classroom atmosphere).
9	Expressing emotions: practical teacher tools to improve student/teacher communication about feelings related to adverse situations.	E-learning discussion of the results of implementing teacher practices based on expressing emotions; intended to make teaching feel present despite the use of e-leaning environments given current circumstances.
10	Using the power of positive emotions in difficult times to develop the teaching profession.	Breakdown of blended learning to design and deliver curricula based on wholesome and purposeful teaching/learning experiences to promote well-being in the educational community.
11	EI strategies and their effectiveness in frontline teacher work in the schools: innovative and successful teaching methodologies and educational plans.	Online stocktaking of the influence of EI strategies on motivation, use of cognitive and metacognitive learning approaches, performance, and overall learning, and their impact on developing educational policies and plans.
12	EI in teachers: training, strategies, and resources to promote positive emotions in the classroom to face and solve challenges.	Online brainstorming of teachers’ key EI experiences/achievements and their adaptive effects on learning and performance.
13	Psychological well-being in the teaching community during adverse and health emergency circumstances: strategies and resources.	Discussion forum to reflect on key strategies and resources to raise psychological well-being and increase satisfaction with the work environment in adverse circumstances.
14	Teacher commitment and definition of future action plans.	Discussion and debate on real experiences in frontline teacher work after implementing strategies from the teacher training program: theoretical and practical implications and future development needs.

**Table 2:** Student’s *t*-test results for difference in mean scores (before training).

Variables	*t*	gl	Sig.	Difference	SD
Harassment–Social acceptance	−1.18	139	0.24	−0.13	0.11
Overload	−1.36	139	0.18	−0.16	0.12
Irritability–tension–fatigue	−1.46	139	0.15	−0.16	0.11
Energy–joy	−0.88	139	0.38	−0.10	0.12
Fear–anxiety	−1.74	139	0.08	−0.23	0.13
Self-realization–satisfaction	−1.96	139	0.05	−0.25	0.13
PSQ total	−1.43	139	0.15	−0.05	0.04
PSS total	−1.26	139	0.21	−3.02	2.38
Skepticism	−1.27	139	0.21	−1.28	1.01
Fatigue	−1.38	139	0.17	−1.25	0.91
Anxiety	−1.13	139	0.26	−0.98	0.87
Inefficiency	−1.13	139	0.26	−0.98	0.87
RED questionnaire total	−1.24	139	0.22	−4.49	3.62
Emotional exhaustion	−1.09	139	0.27	−2.50	2.28
Depersonalization	−1.43	139	0.15	−1.58	1.12
Personal accomplishment	1.47	139	0.14	2.02	1.37
Intrapersonal intelligence	1.23	139	0.22	2.12	1.73
Interpersonal intelligence	0.72	139	0.47	1.25	1.72
Stress management	1.03	139	0.31	1.41	1.37
Adaptation	1.24	139	0.22	1.47	1.19
Humor	1.05	139	0.29	1.84	1.74
EQ-I total	1.09	139	0.28	1.62	1.49
Age	0.74	139	0.46	0.87	1.17
Teaching experience	0.75	139	0.46	0.86	1.15
Gender	0.76	139	0.94	0.01	0.08

**Table 3:** Results of intra-/intersubject univariate ANOVA.

Area Examined	Source	Type III	df	F	Sig.	Partial η^2^	Ob. Power
**Stress levels** **(Perceived Stress Questionnaire)**							
Harassment–social acceptance	Intra	23.15	1	187.83	0.00	0.57	1.00
	Intra*Inter	18.24	1	147.99	0.00	0.52	1.00
	Error intra	17.13	139				
	Inter	1413.36	1	2342.70	0.00	0.94	1.00
	Condition	9.94	1	16.48	0.00	0.11	0.98
	Error inter	83.86	139				
Overload	Intra	20.08	1	133.21	0.00	0.49	1.00
	Intra*Inter	17.27	1	114.57	0.00	0.45	1.00
	Error intra	20.95	139				
	Inter	1409.23	1	2313.21	0.00	0.94	1.00
	Condition	7.93	1	13.01	0.00	0.09	0.95
	Error inter	84.68	139				
Irritability–tension–fatigue	Intra	21.32	1	167.18	0.00	0.55	1.00
	Intra*Inter	21.22	1	166.33	0.00	0.54	1.00
	Error intra	17.73	139				
	Inter	1415.50	1	2570.14	0.00	0.95	1.00
	Condition	10.46	1	19.00	0.00	0.12	0.99
	Error inter	76.55	139				
Energy–joy	Intra	17.52	1	121.91	0.00	0.47	1.00
	Intra*Inter	17.13	1	119.17	0.00	0.46	1.00
	Error intra	19.98	139				
	Inter	1413.68	1	2277.94	0.00	0.94	1.00
	Condition	10.70	1	17.23	0.00	0.11	0.98
	Error inter	86.26	139				
Fear–anxiety	Intra	17.41	1	86.49	0.00	0.38	1.00
	Intra*Inter	23.26	1	115.55	0.00	0.45	1.00
	Error intra	27.98	139				
	Inter	1439.51	1	1986.80	0.00	0.94	1.00
	Condition	8.51	1	11.74	0.00	0.08	0.93
	Error inter	100.71	139				
Self-realization–satisfaction	Intra	19.00	1	107.03	0.00	0.44	1.00
	Intra*Inter	20.05	1	112.92	0.00	0.45	1.00
	Error intra	24.68	139				
	Inter	1433.03	1	2009.57	0.00	0.94	1.00
	Condition	5.72	1	8.02	0.01	0.05	0.80
	Error inter	99.12	139				
PSQ total	Intra	2.29	1	200.51	0.00	0.59	1.00
	Intra*Inter	2.15	1	188.18	0.00	0.58	1.00
	Error Intra	1.59	139				
	Inter	48.21	1	763.83	0.00	0.85	1.00
	Condition	1.04	1	16.46	0.00	0.11	0.98
	Error inter	8.77	139				
**Stress levels** **(Perceived Stress Scale)**							
PSS total	Intra	11,816.55	1	248.92	0.00	0.64	1.00
	Intra*Inter	12,840.51	1	270.49	0.00	0.66	1.00
	Error intra	6598.42	139				
	Inter	18,0340.59	1	683.03	0.00	0.83	1.00
	Condition	7739.95	1	29.32	0.00	0.17	1.00
	Error inter	36,700.04	139				
**ICT competency** **(RED questionnaire)**							
Skepticism	Intra	949.57	1	212.56	0.00	0.61	1.00
	Intra*Inter	857.19	1	191.88	0.00	0.58	1.00
	Error intra	620.95	139				
	Inter	21,107.47	1	372.52	0.00	0.73	1.00
	Condition	342.30	1	6.04	0.02	0.04	0.69
	Error inter	7876.02	139				
Fatigue	Intra	778.60	1	219.21	0.00	0.61	1.00
	Intra*Inter	909.04	1	255.94	0.00	0.65	1.00
	Error intra	493.70	139				
	Inter	20,503.90	1	403.65	0.00	0.74	1.00
	Condition	386.02	1	7.60	0.01	0.05	0.78
	Error inter	7060.62	139				
Anxiety	Intra	271.20	1	65.52	0.00	0.32	1.00
	Intra*Inter	1461.33	1	353.06	.00	0.72	1.00
	Error intra	575.32	139				
	Inter	23,137.47	1	592.80	0.00	0.81	1.00
	Condition	900.30	1	23.07	0.00	0.14	1.00
	Error inter	5425.28	139				
Inefficiency	Intra	277.16	1	65.83	0.00	0.32	1.00
	Intra*Inter	1475.11	1	350.37	0.00	0.72	1.00
	Error intra	585.21	139				
	Inter	23,082.77	1	588.25	0.00	0.81	1.00
	Condition	911.13	1	23.22	0.00	0.14	1.00
	Error inter	5454.34	139				
RED questionnaire total	Intra	8433.61	1	163.86	0.00	0.54	1.00
	Intra*Inter	18,513.04	1	359.69	0.00	0.72	1.00
	Error intra	7154.24	139				
	Inter	351,075.34	1	482.47	0.00	0.78	1.00
	Condition	9670.52	1	13.29	0.00	0.09	0.95
	Error inter	101,144.47	139				
**Burnout levels** **(Maslach Burnout Inventory)**							
Emotional exhaustion	Intra	4088.17	1	231.10	0.00	0.62	1.00
	Intra*Inter	4225.40	1	238.85	0.00	0.63	1.00
	Error intra	2458.97	139				
	Inter	107,684.19	1	376.51	0.00	0.73	1.00
	Condition	1936.07	1	6.77	0.01	0.05	0.73
	Error inter	39755.41	139				
Depersonalization	Intra	663.43	1	135.98	0.00	0.50	1.00
	Intra*Inter	2168.79	1	444.54	0.00	0.76	1.00
	Error intra	678.15	139				
	Inter	35,342.12	1	535.69	0.00	0.79	1.00
	Condition	1098.58	1	16.65	0.00	0.11	0.98
	Error inter	9170.58	139				
Personal accomplishment							
	Intra	1201.03	1	126.30	0.00	0.48	1.00
	Intra*Inter	1120.47	1	117.82	0.00	0.46	1.00
	Error intra	1321.84	139				
	Inter	376,330.30	1	3922.13	0.00	0.97	1.00
	Condition	272.20	1	2.84	0.09	0.02	0.39
	Error inter	13,337.13	139				
**Emotional Intelligence Scores** **(Emotional Quotient Inventory)**							
Intrapersonal intelligence	Intra	1972.74	1	60.27	0.00	0.30	1.00
	Intra*Inter	4379.09	1	133.79	0.00	0.49	1.00
	Error intra	4549.55	139				
	Inter	355,852.16	1	3431.15	0.00	0.96	1.00
	Condition	2332.50	1	22.49	0.00	0.14	1.00
	Error inter	14,415.99	139				
Interpersonal intelligence	Intra	2083.50	1	69.78	0.00	0.33	1.00
	Intra*Inter	4002.53	1	134.06	0.00	0.49	1.00
	Error intra	4150.13	139				
	Inter	359,951.42	1	3237.00	0.00	0.96	1.00
	Condition	2786.40	1	25.06	0.00	0.15	1.00
	Error inter	15,456.67	139				
Stress management	Intra	3244.44	1	166.65	0.00	0.55	1.00
	Intra*Inter	5146.92	1	264.37	0.00	0.66	1.00
	Error intra	2706.19	139				
	Inter	175,000.07	1	2782.86	0.00	0.95	1.00
	Condition	3587.34	1	57.05	0.00	0.29	1.00
	Error inter	8741.03	139				
Adaptation	Intra	2181.24	1	149.90	0.00	0.52	1.00
	Intra*Inter	3765.98	1	258.81	0.00	0.65	1.00
	Error intra	2022.64	139				
	Inter	134,588.56	1	2796.80	0.00	0.95	1.00
	Condition	2399.97	1	49.87	0.00	0.26	1.00
	Error inter	6689.02	139				
Humor	Intra	4565.04	1	148.18	0.00	0.52	1.00
	Intra*Inter	7978.82	1	259.00	0.00	0.65	1.00
	Error intra	4282.12	139				
	Inter	278,026.74	1	2693.19	0.00	0.95	1.00
	Condition	5459.25	1	52.88	0.00	0.28	1.00
	Error inter	14349.40	139				
EQ-I total	Intra	2730.89	1	123.44	0.00	0.47	1.00
	Intra*Inter	4952.62	1	223.87	0.00	0.62	1.00
	Error intra	3075.04	139				
	Inter	251,796.94	1	3329.37	0.00	0.96	1.00
	Condition	3222.90	1	42.62	0.00	0.24	1.00
	Error inter	105,12.43	139				

## Appendix A. Description about Teacher Training Evaluation Survey

A survey was designed ad-hoc by the authors to determine how satisfied teachers were with the 14-week training program after their participation. The survey consisted of 15 statements rated on a 5-point Likert scale from 1 (“totally disagree”) to 5 (“totally agree”). A comment box was also included to allow teachers to share remarks or suggestions. The 15 items were as follows:

1. The teacher training program effectively improves teachers’ ability to cope with stress.
2. The teacher training program effectively prevents burnout among teachers.
3. The teacher training program effectively improves teachers’ ICT competency.
4. The teacher training program effectively trains teachers to apply EI competencies to educational management.
5. The teacher training program is helpful for developing innovative and successful teaching methodologies and educational plans as a strategic response to address the pandemic.
6. The objectives were appropriate for the planned duration and the established work schedule.
7. The methods used were effective.
8. The transfer of knowledge to my teaching practice was understandable.
9. The knowledge I have gained will positively affect my methods in the classroom.
10. The 14-week teacher training program met my expectations.
11. My motivation and interest during the 14-week teacher training program were high.
12. The teacher training program fosters good EI in teachers to promote psychological well-being in the teaching community.
13. The teacher training program effectively expands teachers’ commitment to quality education and addresses future gaps.
14. The training was engaging and beneficial for improving academic achievement based on positive emotions in primary school.
15. This teacher training program was a good impetus for me to improve my performance as a teacher.
